# Translation of the Dimensional Apathy Scale to Brazilian Portuguese to assess people living with HIV

**DOI:** 10.1590/1980-5764-DN-2025-0343

**Published:** 2026-03-23

**Authors:** Fernando Nonato de Carvalho Fagundes, Aleida Nazareth Soares, Ratko Radakovic, Sindy Sthefany Sousa Silva, Adriana Silvina Pagano, Antônio Lúcio Teixeira, Alexandre Sampaio Moura

**Affiliations:** 1 Faculdade Santa Casa Belo Horizonte, Belo Horizonte MG, Brazil.; 2 Universidade José do Rosário Vellano, Belo Horizonte MG, Brazil.; 3 University of East Anglia, Norwich Medical School, Norwich, UK.; 4 University of Edinburgh, Euan MacDonald Centre for Motor Neuron Disease Research, Edinburgh, UK.; 5 University of Edinburgh, Alzheimer Scotland Dementia Research Centre, Edinburgh, UK.; 6 Cambridgeshire and Peterborough NHS Foundation Trust, Cambridge, UK.; 7 Universidade Federal de Minas Gerais, Faculdade de Letras, Belo Horizonte MG, Brazil.; 8 University of Texas Health Science Center, Biggs Institute, San Antonio, Texas, United States of America.

**Keywords:** Translating, Psychometrics, Apathy, HIV, Tradução, Psicometria, Apatia, HIV

## Abstract

Neurocognitive and behavioral disorders are common among people living with HIV (PLHIV) and can be associated with apathy. The use of a valid and reliable instrument to identify apathy among PLHIV could allow early diagnosis and proper management of this condition.

**Objective:**

The aim of this paper was to translate, cross-culturally adapt, and validate the caregiver-rated Dimensional Apathy Scale (DAS) in PLHIV in Brazil.

**Methods:**

We followed five steps: translation, back translation, cultural adaptation, pretest, and test–retest. A pretest (n=20) and test-retest (n=80) were conducted with PLHIV from an HIV Referral Center in Brazil between August 2022 and March 2023. We determined the content validity index (CVI), Cronbach’s alpha, and the intraclass correlation coefficient (ICC). To assess convergent and discriminant validity, apathy scores were correlated with cognitive function, as assessed by the International HIV Dementia Scale (IHDS), and depressive symptoms, as assessed by the Patient Health Questionnaire (PHQ-9).

**Results:**

Participants were predominantly male (82.5%), with a mean age of 41.7 (±13.4) years and a mean duration of infection of 8.1 (±1.9) years. The mean CVI of the Brazilian DAS version (DAS-Br) was 0.97. The translated version showed acceptable internal consistency (Cronbach’s alpha: 0.727 (95%CI 0.632–0.806)) and good test–retest reliability (ICC 0.888 (95%CI 0.823–0.929). There was a moderate correlation between DAS-Br scores and depressive symptoms (r=0.425, p<0.01) and a nonsignificant correlation with cognition (r=0.167; p>0.05).

**Conclusion:**

The translation and cross-cultural adaptation of the DAS into Brazilian Portuguese resulted in a valid and reliable instrument for assessing apathy in PLHIV.

## RESUMO.

Distúrbios neurocognitivos e comportamentais são comuns em pessoas vivendo com HIV (PVHIV) e podem estar associados à apatia. O uso de um instrumento válido e confiável para identificar a apatia em PVHIV pode permitir o diagnóstico precoce e o manejo adequado dessa condição.

**Objetivo:**

Traduzir, adaptar transculturalmente e validar a Escala Dimensional de Apatia (DAS), preenchida por cuidadores, em PVHIV no Brasil.

**Métodos:**

Seguimos cinco etapas: tradução, retrotradução, adaptação cultural, pré-teste e teste-reteste. O pré-teste (n=20) e o teste-reteste (n=80) foram realizados com PVHIV de um Centro de Referência em HIV no Brasil entre agosto de 2022 e março de 2023. Determinamos o índice de validade de conteúdo (IVC), o alfa de Cronbach e o coeficiente de correlação intraclasse (CCI). Para avaliar a validade convergente e discriminante, os escores de apatia foram correlacionados com a função cognitiva, avaliada pela Escala Internacional de Demência por HIV (IHDS), e com os sintomas depressivos, avaliados pelo Questionário de Saúde do Paciente (PHQ-9).

**Resultados:**

Os participantes eram predominantemente do sexo masculino (82,5%), com idade média de 41,7 (±13,4) anos e tempo médio de infecção de 8,1 (±1,9) anos. O IVC médio da versão brasileira da DAS (DAS-Br) foi de 0,97. A versão traduzida apresentou consistência interna aceitável (alfa de Cronbach: 0,727 (IC95% 0.632–0.806)) e boa confiabilidade teste-reteste (CCI 0,888 (IC95% 0.823–0.929). Houve uma correlação moderada entre os escores da DAS-Br e os sintomas depressivos (r=0,425, p<0,01) e uma correlação não significativa com a cognição (r=0,167; p>0,05).

**Conclusão:**

A tradução e a adaptação transcultural da DAS para o português brasileiro resultaram em um instrumento válido e confiável para avaliar a apatia em PVHIV.

## INTRODUCTION

 Apathy is a clinical syndrome characterized by reduced motivation for goal-directed behaviors and cognitive activity accompanied by blunted affect^1^. Apathy has been associated with a series of neurodegenerative and psychiatric conditions and is frequently observed in HIV-infected patients^2,3^. Some studies estimate the prevalence of apathy in people living with HIV (PLHIV) at up to 50%. Apathy in PLHIV has been related to adverse outcomes for everyday functions, cognitive performance, and quality of life and has been associated with other neuropsychiatric symptoms (e.g., depression) and nonadherence to antiretroviral therapy^3-6^. 

 The Dimensional Apathy Scale (DAS) is a multidimensional apathy measure quantifying different apathy subtypes called "Executive" (lack of motivation for planning, organization, and attention), "Emotional" (emotional indifference, blunting, or flatness), and "Initiation" (lack of motivation for self-generation of thoughts or actions). This tool was initially designed in English and has already been translated and validated for use in Japanese, Spanish, Italian, and French populations^7-10^. However, this tool has not yet been translated and validated into Brazilian Portuguese and has not been used to explore apathy profiles in PLHIV. There is a Brazilian version of the Apathy Scale, but it has only been tested in people with dementia. 

 This study aimed to translate, cross-culturally adapt, and validate the DAS instrument for Brazilian Portuguese in PLHIV. 

## METHODS

### Study design

 The study followed the five steps proposed by Ramada and Abrahams^11^: forward translation of the caregiver-rated DAS into Portuguese, back translation into English, cultural adaptation, pretest, and test–retest. The study was conducted at a referral HIV care center for PLHIV in Belo Horizonte, Brazil, between August 2022 and March 2023. Sociodemographic and clinical information was obtained through medical chart review. 

 The study was approved by the Institutional Review Boards of Santa Casa de Belo Horizonte and Unifenas (CAAE 51783521.1.0000.5138). All participants provided informed consent. 

### Participants

 Individuals aged ≥18 years, with confirmed HIV infection, who agreed to participate and had at least 4 years of education (elementary school) were included in the study. The latter criterion was set to favor the inclusion of people with a better understanding of the interview process. Patients were excluded from the study if they had any documented severe psychiatric (e.g., psychotic) disorder or intellectual disability. 

### Procedures

#### Translation and back translation

 Two translators (T1 and T2), native Brazilians, initially translated the questionnaire forward. One translator was familiar with the instrument’s objectives and had prior experience with the topic, while the other was unfamiliar with the scale’s objectives and had no experience with the topic. 

 The translators (T1 and T2) discussed their different versions, and a final consensus version was established. The researchers did not need to intervene in this step, as there was no significant incongruence between the translators (T1-2). 

 Then, two bilingual translators (BT1 and BT2), whose primary language is English and who have excellent knowledge of the Portuguese language, back-translated the first translated version. After concluding this step, the two back-translated versions were compared to the original English version of the scale. Upon verifying that there was no relevant disagreement, the T1-2 version in Brazilian Portuguese was used in the subsequent steps. 

 The researchers then adjusted the translated version so that the items could be answered directly by patients rather than by their caregivers, as the original version of the scale had proposed. 

#### Cultural adequacy and adaptation

 Each of the six expert committee members received the original version of the DAS and the final translated version (T1-2). 

 They were requested to evaluate each item of the translated instrument, compared to the original version, and provide a score according to the following scale:

1. One star: need for complete retranslation;
2. Two stars: need for partial retranslation with significant editing;
3. Three stars: need for partial retranslation with optional editing to improve style;
4. Four stars: no need for any editing or retranslation.

The judges were also asked to comment on the translated items, providing suggestions for improving the instrument. 

### Pretest

 The pretest was conducted with 20 PLHIV. Participants were instructed to read all items and report their impressions regarding the clarity and ambiguity of each item. At this stage, they were not asked to complete the questionnaire. 

### Test and retest

 In total, 92 eligible patients answered the Brazilian version of DAS (DAS-Br) and the PHQ-9. A trained researcher assessed cognitive function using the IHDS. Notably, 15 days after the test, all participants received a phone call, and 80 answered again to the DAS-Br. Our group chose to work with the version in an interview format to monitor patients’ understanding of the translated version since the early stages of the process and to create an instrument that can be used by clinicians who manage PLHIV. 

### Measures

 All participants completed the interview-based DAS-Br, with higher scores indicating greater apathy. Participants also completed the Patient Health Questionnaire (PHQ-9) as a measure of depressive symptoms, with higher scores indicating more depressive symptoms. Further, the International HIV Dementia Scale (IHDS) was used to quantify cognitive functioning, with lower scores indicating poorer performance^12,13^. The cut-off for depression was >9 points in the PHQ-9 (indicative of moderate or severe depression), and the cut-off for dementia was ≤10 points on the IHDS. Apathy was defined according to the cut-off points of the original version of DAS (≥14 points for the executive subscale, ≥15 points for the emotional subscale, ≥16 points for the initiation subscale, and ≥39 points for the overall DAS score). 

### Data analysis

 Considering a significance level of 5%, a power of 80%, and an intraclass correlation coefficient of 0.30, the sample size was estimated at 82 respondents^14^. 

 Reliability was assessed using intraclass correlation (ICC), while internal content validity was evaluated using the content validity index (CVI). For instruments previously constructed and validated in other cultures, a minimum CVI of 0.78 should be anticipated. Additionally, a recommended threshold above 0.70 for ICC indicates good test–retest reliability^15^. Cronbach’s standardized alpha was utilized to estimate internal consistency, with values greater than 0.70 indicating good consistency^11^. 

 Spearman’s r was used to assess the convergence and divergence of the DAS-Br score with the IHDS and PHQ-9. 

 All statistical analyses were performed using IBM SPSS Statistics for Windows, version 23.0 (IBM Corp., Armonk, NY, USA), applying two-tailed tests with a significance level set at p < 0.05. 

## RESULTS

### Translation and cultural adaptation

 After the expert committee’s first round of analysis, the minimum IVC threshold of 80% was not reached for the instructions on how to fill out the questionnaire or for item 16, which were subsequently revised. These items were adjusted, and in the second Delphi round, all items reached the IVC threshold (mean IVC: 0.97). The final Brazilian DAS version (DAS-Br) is available as Supplementary Material (available at https://www.demneuropsy.org/wp-content/uploads/2025/12/DN-2025.0343-Supplementary-Material.docx). 

### Pretest

 The pretest was conducted with 20 patients (95% male, 60% homosexual/gay/lesbian, mean (±SD) age, and schooling years: 37 (±13.20) and 13 (±2.56), respectively). Items 15 and 24 received more than 15% of requests for clarification, and minor changes were made to improve them. 

### Internal consistency and test-retest reliability

 In total, 92 patients were included in this step of the study. The sociodemographic and clinical characteristics of these participants are presented in Table 1. Notably, 10 (10.9%) patients were positively screened for apathy, 32 patients (36.3%) were positively screened for cognitive impairment (IDHS ≤10 points), and 22 (23.9%) were positively screened for depressive symptoms (PHQ-9 >9 points). 

**Table 1:** Participants characteristics (n=92).

Characteristics		n=92 (%)
Age (years) (mean±SD)		41.4 (±13.40)
Sex		
	Male	78 (84.8)
	Female	14 (15.2)
Years of education (mean±SD)		12.41 (±3.77)
Comorbidities		
	Syphilis	39 (42.4)
	Chronic hepatitis B	1 (1.1)
	Chronic hepatitis C	3 (3.3)
	Systemic arterial hypertension	13 (14.1)
	Diabetes mellitus	6 (6.5)
	Chronic kidney disease	3 (3.2)
	Depression	10 (10.9)
	Use of illicit drugs in the last 30 days	15 (16.3)
	Excessive alcohol intake (last 30 days)	25 (27.2)
	Myocardial infarction	1 (1.1)
	Kaposi’s sarcoma	1 (1.1)
Nadir CD4 T cell count (/mm^3^)		532.37 (±314.73)
Time since HIV diagnosis (years)		8.12 (±1.92)
Current ART		
	TDF+3TC+DTG	63 (68.5)
	TDF+3TC+EFV	19 (20.6)
	Other	10 (10.9)
Regular use of ART (previous 6 months)		84 (91.3)

Abbreviations: SD, standard deviation, HIV: human immunodeficiency virus; ART, antiretroviral therapy; TDF, tenofovir difuramate; 3TC, lamivudine; DTG, dolutegravir; EFV, efavirenz.

 The overall Cronbach’s alpha value for the DAS-BR was 0.727 (0.632–0.806). The values for the emotional, executive, and cognitive-behavioral initiation subscales were 0.418 (0.218–0.600), 0.748 (0.661–0.820), and 0.669 (0.556–0.762), respectively. No translated item from the original version of DAS needed to be suppressed. The Cronbach’s alpha values of the DAS-Br after removing each item are presented as supplementary material. 

 In total, 80 patients (87.0%) answered the retest and were included in the test-retest analysis. The overall ICC estimate for DAS-Br was 0.888 (0.823–0.929). There was no statistically significant difference between test and retest scores. 

### Convergent and divergent validity analysis

 A moderate correlation was found between the overall scores of the DAS-Br and PHQ-9 (r=0.425, p<0.01). The executive and initiation subscales of DAS-Br showed a weak positive correlation with the PHQ-9, and no association was found between PHQ-9 scores and the emotional domain. The correlation between DAS-Br and IHDS scores was positive but nonsignificant (r=0.167; p>0.05) (Table 2). 

**Table 2:** Correlations between Dimensional Apathy Scale-Br scores (overall and domain-specific) and Patient Health Questionnaire-9 and International HIV Dementia Scale scores.

DAS-Br (n=80)	DAS-Br executive	DAS-Br emotional	DAS-Br initiation	PHQ-9	IHDS
Executive domain	1.000			0.372† [0.167–0.549]	
Emotional domain	0.100 [-0.122–0.313]	1.000		0.090 [-0.133–0.304]	
Initiation domain	0.254* [0.036–0.449]	0.300 [0.086–0.488]	1.000	0.396† [0.193–0.567]	
Overall score				0.425† [0.225–0.589]	0.167 [-0.055–0.374]

Abbreviations: DAS: Dimensional Apathy Scale; PHQ-9, Patient Health Questionnaire; IHDS, International HIV Dementia Scale; HIV: HIV: human immunodeficiency virus.

Notes: Data are presented as Spearman’s correlation coefficients (p) and corresponding 95% confidence intervals [95%CI].

*Spearman’s correlation is significant at the 0.05 level; †Spearman’s correlation is significant at the 0.01 level.

Table 3 presents the ICC values related to temporal stability, specifically the variation in responses from the same patient to the questionnaire after at least 15 days following test application ("test-retest"). There was no statistically significant difference between the medians of the test and retest by subscale or in the overall score. The overall ICC value for the DAS-Br was 0.888 (0.823–0.929). 

**Table 3:** Evaluation of internal consistency and temporal stability of the Portuguese version of the scale.

Subscale (no. of items)	Median test (p25–p75)	Median retest (p25–p75)	p-value*	ICC (95%CI)	Cronbach’s Alpha (95%CI)
Executive (8)	8.0 (5.0–13.0)	9.0 (4.0–13.0)	0.350	0.911 (0.861–0.943)	0.748 (0.661–0.820)
Emotional (8)	8.0 (5.0–10.5)	6.0 (5.0–10.0)	0.093	0.705 (0.540–0.811)	0.418 (0.218–0.600)
Cognitive-behavioral initiation (8)	6.0 (3.0–9.0)	6.0 (3.0–9.0)	0.251	0.813 (0.708–0.880)	0.669 (0.556–0.762)
Overall	23.0 (17.0–30.5)	22.0 (15.0–27.0)	0.052	0.888 (0.823–0.929)	0.727 (0.632–0.806)

Abbreviations: p25, 25th percentile; p75, 75th percentile; CI, confidence interval; ICC, intraclass correlation coefficient; p, probability of significance or p-value.

Notes: Dimensional of Apathy (n = 80).

* Wilcoxon Test.

### Preliminary apathy profile in PLHIV

 In total, 27 (29.3%) patients displayed one or more apathy subtype impairments. Notably, 22 (23.9%) participants displayed executive apathy, six (6.5%) emotional apathy, and two (2.2%) initiation apathy. Only four participants had impairments in more than one apathy subtype. Specifically, two had executive and emotional apathy, one had executive and initiation apathy, and one had emotional and initiation apathy. 

## DISCUSSION

 The results of this study show that a robustly translated Brazilian Portuguese version of the Dimensional Apathy Scale (DAS) is a reliable and valid instrument for use in PLHIV. 

 The internal consistency of the DAS-Br was similar to that reported for the original DAS and its translations into French, Japanese, and Spanish. Cronbach’s alpha values greater than 0.80 were obtained only for the Italian version of the instrument^7-10^. Furthermore, the test–retest reliability of DAS-Br was also high, like that observed in the Spanish version of the instrument^8^. 

 When examining intercorrelations of apathy subtypes, this study also found a similar pattern to previous translations^7^, with some variability regarding the degree of correlation between subtypes^8,16^. Our study found associations between the executive and initiation subscales and PHQ-9 scores. Previous studies have also found associations between the executive and initiation subscales and depression^10^. Notably, we did not find significant associations between depression and emotional subscale scores, in line with previous translation findings^8^. Future research should better explore the interplay between apathy subtypes and depression. 

 The observation of a lower Cronbach’s alpha for the emotional subscale, which falls below the acceptable threshold for this measure, raises questions about the internal consistency of the scale or the homogeneity of its items in this specific domain. While the inherent multidimensionality of the apathy construct, which encompasses emotional, behavioral, and cognitive aspects, may be a plausible explanation, given that homogeneity is inherently lower in complex, multifaceted constructs, it is essential to move beyond this hypothesis. For example, cultural differences in how emotions are expressed and perceived may influence how participants interpret and respond to the items, leading to greater non-trait-related variance. Alternatively, limited variability in the sample’s responses (floor or ceiling effects) could have artificially lowered the coefficient. Our study’s overall prevalence of apathy is different from previous reports, showing a prevalence of 37% among PLHIV^5^. The higher prevalence of neuropsychiatric disorders and cognitive impairment in other studies might be explained by differences in sociodemographic (e.g., lower educational level) and clinical characteristics. The current study found that executive apathy was observed in almost a quarter of this group of PLHIV. These findings are preliminary, and future research should investigate the apathy profiles in PLHIV and possible correlates of amotivation. 

 Unlike the French study with people with schizophrenia, we found a higher prevalence of the emotional subscale than the initiation one^10^. On the other hand, the Spanish study found that the subtype of apathy initiation was more common among people with amyotrophic lateral sclerosis^8^. 

 This study has limitations that must be acknowledged. First, it was conducted in a single HIV referral center. Nonetheless, the sociodemographic characteristics of individuals in our study align with the Brazilian profile of PLHIV^17^. Additionally, this study used the original English language-based cutoffs in determining apathy profiles. Future research should aim to determine culturally specific norms and cutoffs to facilitate the interpretation of apathy profiles for the Brazilian population. Furthermore, the assessment was conducted using an interview format to monitor patients’ understanding of the translated version from the beginning of the work (pretest) and to create an instrument that can be used by doctors who treat people living with HIV. This could have introduced a potential response bias by making them choose options that would be considered more socially acceptable. Finally, the construct validity could have been more robustly evaluated with the inclusion of a Confirmatory Factor Analysis (CFA). The absence of CFA prevents a direct and rigorous test of the adequacy of the three-factor model of the Dimensional Apathy Scale (DAS-Br), leaving the three-factor structure inferred without the necessary statistical validation to confirm whether the observed data fit the proposed theoretical model. 

 In conclusion, DAS-Br is the first multidimensional apathy measure translated into Brazilian Portuguese. It is a reliable and valid instrument for assessing apathy subtypes among PLHIV and can be used in broader clinical practice and scientific research in Brazil. 

## Data Availability

The datasets generated and/or analyzed during the current study are available from the corresponding author upon reasonable request.
