# Supplementary material for: Translation of the Dimensional Apathy Scale to Brazilian Portuguese to assess people living with HIV
Source: Dement Neuropsychol. 2026 Mar 23;20:e20250343. doi: 10.1590/1980-5764-DN-2025-0343 (PMC13008347; doi:10.1590/1980-5764-DN-2025-0343)
Supplement: Supplementary material 1 [file 1980-5764-dn-20-e20250343-md01.docx]

**Supplementary Material**

**Cronbach's Alpha of the DAS-Br if each instrument item is removed.**

| **Subscale and items** | **Cronbach's alpha if the item is removed** |
| --- | --- |
| ***Executive*** |  |
| 1 | 0.727 |
| 6 | 0.713 |
| 10 | 0.706 |
| 11 | 0.696 |
| 17 | 0.733 |
| 19 | 0.710 |
| 21 | 0.699 |
| 23 | 0.710 |
| ***Emotional*** |  |
| 3 | 0.726 |
| 5 | 0.713 |
| 7 | 0.733 |
| 9 | 0.717 |
| 12 | 0.728 |
| 15 | 0.743 |
| 20 | 0.722 |
| 24 | 0.723 |
| ***Initiation*** |  |
| 2 | 0.713 |
| 4 | 0.719 |
| 8 | 0.722 |
| 13 | 0.714 |
| 14 | 0.724 |
| 16 | 0.701 |
| 18 | 0.718 |
| 22 | 0.715 |
